# Adrenal insufficiency following cerebellar intracranial hemorrhage: a case report

**DOI:** 10.3389/fneur.2024.1332604

**Published:** 2024-03-12

**Authors:** Yu-Yang Lu, Chien-Ming Lin, Shang-Lin Chiang

**Affiliations:** ^1^Department of Pediatrics, Tri-Service General Hospital, National Defense Medical Center, Taipei, Taiwan; ^2^Department of Physical Medicine and Rehabilitation, Tri-Service General Hospital, National Defense Medical Center, Taipei, Taiwan

**Keywords:** cerebellar hemorrhage, corticosteroids, adrenal insufficiency, rehabilitation, case report

## Abstract

This report presents a case of a previously healthy 58 years-old man who had suffered from persistent weakness and dizziness after a cerebellar intracranial hemorrhage (ICH). Endocrine function tests revealed low levels of plasma cortisol (3.05 μg/dL; normal range: 5–25 μg/dL) and adrenocorticotropic hormone (ACTH) (6.0 pg/mL; normal range: 10–60 pg/mL). The subsequent ACTH stimulation test suggested partial or recent hypopituitarism, resulting in adrenal gland atrophy and a subnormal cortisol response. Ultimately, the dizziness was found to be caused by undiagnosed adrenal insufficiency, which was detected when a hypotensive fainting incident occurred during rehabilitation. The symptoms improved significantly with oral prednisone supplementation. Notably, the duration of impaired hypothalamic-pituitary-adrenal axis may last as long as a year. This case highlights that adrenal insufficiency can easily be overlooked since its symptoms are similar to those commonly seen with cerebellar stroke alone. Physicians must be aware of the symptoms of adrenal insufficiency in patients with brain insults and conduct the appropriate endocrine tests to clarify the underlying comorbidity.

## Introduction

Dizziness is a common symptom among patients suffering from cerebellar intracranial hemorrhage (ICH). However, dizziness is a nonspecific symptom that may also be caused by endocrinological disorders, such as adrenal insufficiency. On the other hand, critical illness can result in suppressed plasma adrenocorticotropic hormone (ACTH) levels, a condition known as critical illness-related corticosteroid insufficiency (CIRCI) (1), making dizziness an overlapping symptom associated with both cerebellar ICH and secondary adrenal insufficiency.

Cortisol levels are often increased during critical conditions. Extended periods of elevated cortisol may affect the hypothalamic-pituitary-adrenal (HPA) axis by suppressing expression levels of ACTH in the pituitary gland via negative feedback mechanisms, thereby suppressing the production and release of plasma ACTH. Over time, the absence of ACTH signaling to the adrenal gland results in a loss of integrity and function of the HPA axis (2, 3). This may cause adrenal insufficiency, which manifests in variable signs and symptoms (Table 1) including weakness, fatigue, dizziness, and mimicking ICH sequelae, among many others (2, 4, 5).

**Table 1:** Clinical manifestations of chronic adrenal insufficiency (table from UpToDate: Diagnosis of adrenal insufficiency in adults).

Symptom	Frequency (%)
Weakness, tiredness, fatigue	100
Anorexia	100
Gastrointestinal symptoms	92
Nausea	86
Vomiting	75
Constipation	33
Abdominal pain	31
Diarrhea	16
Salt craving	16
Postural dizziness	12
Muscle or joint pains	6 to 13
**Sign**	
Weight loss	100
Hyperpigmentation	94
Hypotension (systolic BP <110 mmHg)	88 to 94
Vitiligo	10 to 20
Auricular calcification	5

This article presents a previously healthy 58 years-old man who developed adrenal insufficiency, characterized by persistent dizziness, while undergoing rehabilitation after cerebellar ICH. This was initially undiagnosed at the time of the ICH, and took him 1 year to recover. Case series guidelines were adhered to (6).

## Case

**Figure 1:**
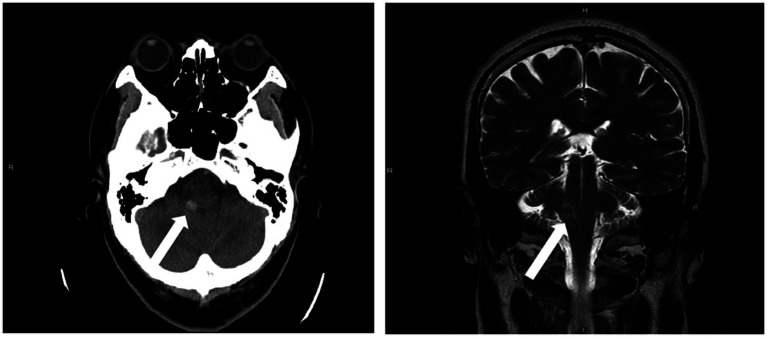
A 1 cm intracranial hemorrhage (ICH) in the right cerebellar peduncle was detected on a non-contrast brain CT scan (left) and brain MRI (right).

This case presents a 58 years-old male—in his usual state of health until 1 year ago—suffering from unsteady gait, nausea, and vomiting when working. He possessed no significant past medical history and denied taking regular medications. Despite these symptoms, he did not immediately seek medical treatment. After enduring the symptoms for another 6 days, the patient presented at the emergency department. Upon examination, his Glasgow Coma Scale (GCS) score was 15, with normal pupil size and light reflex, but with a decreased left-sided extremity muscle power of 4 and tilting to the right when standing. Laboratory tests showed no significant abnormalities. However, a non-contrast brain computed tomography (CT) revealed a 1 cm ICH in his right cerebellar peduncle (Figure 1). Upon discovery of these findings, he was soon admitted to the intensive care unit for close monitoring.

Fortunately, the patient did not exhibit any fatal clinical signs. Upon transferal to the ordinary ward, his modified Rankin Scale was 3, with sustained ataxia and dysphagia. The patient then began a rehabilitation program, including physical therapy, occupational therapy, and speech therapy.

In the sixth month after the discovery of the cerebellar ICH, the patient was transferred to our hospital due to persistent weakness and dizziness, particularly in the recent month. The symptoms of dizziness and weakness had limited his participation and mobility during rehabilitation. As the patient was informed that the intractable symptoms might be remnants of the cerebellar ICH, the patient did not undergo further examination. During previous hospitalizations, he received only partial relief from medications, including diphenidol and meclizine, leaving the symptoms unresolved. Upon acceptance examination, the motor status of his extremities were all rated as Brunnstrom’s stage V. He possessed good static and dynamic sitting balance and could stand up with fair dynamic balance and walk with the aid of a walker, but still needed assistance with maintaining personal hygiene.

However, during the first week of hospitalization, the patient fainted while receiving physical therapy due to a transient loss of consciousness. Immediate blood sugar tested at 101 mg/dL, with body temperature measured as afebrile, and a blood pressure measurement of 79/50 mmHg. Laboratory examination revealed no obvious abnormalities in electrolytes or biochemistry (Na: 138 mmol/L, K: 4.6 mmol/L, Hb: 14.6 g/dL, WBC: 3380/μL). Furthermore, endocrine function tests conducted the next morning resulted in a low level of plasma cortisol at 3.05 μg/dL (normal range: 5–25 μg/dL) and a low level of plasma ACTH at 6.0 pg/mL (normal range: 10–60 pg/mL), despite normal free T4 (1.10 ng/dL), TSH (3.78 μIU/mL), and aldosterone level (88.9 pg/mL).

**Figure 2:**
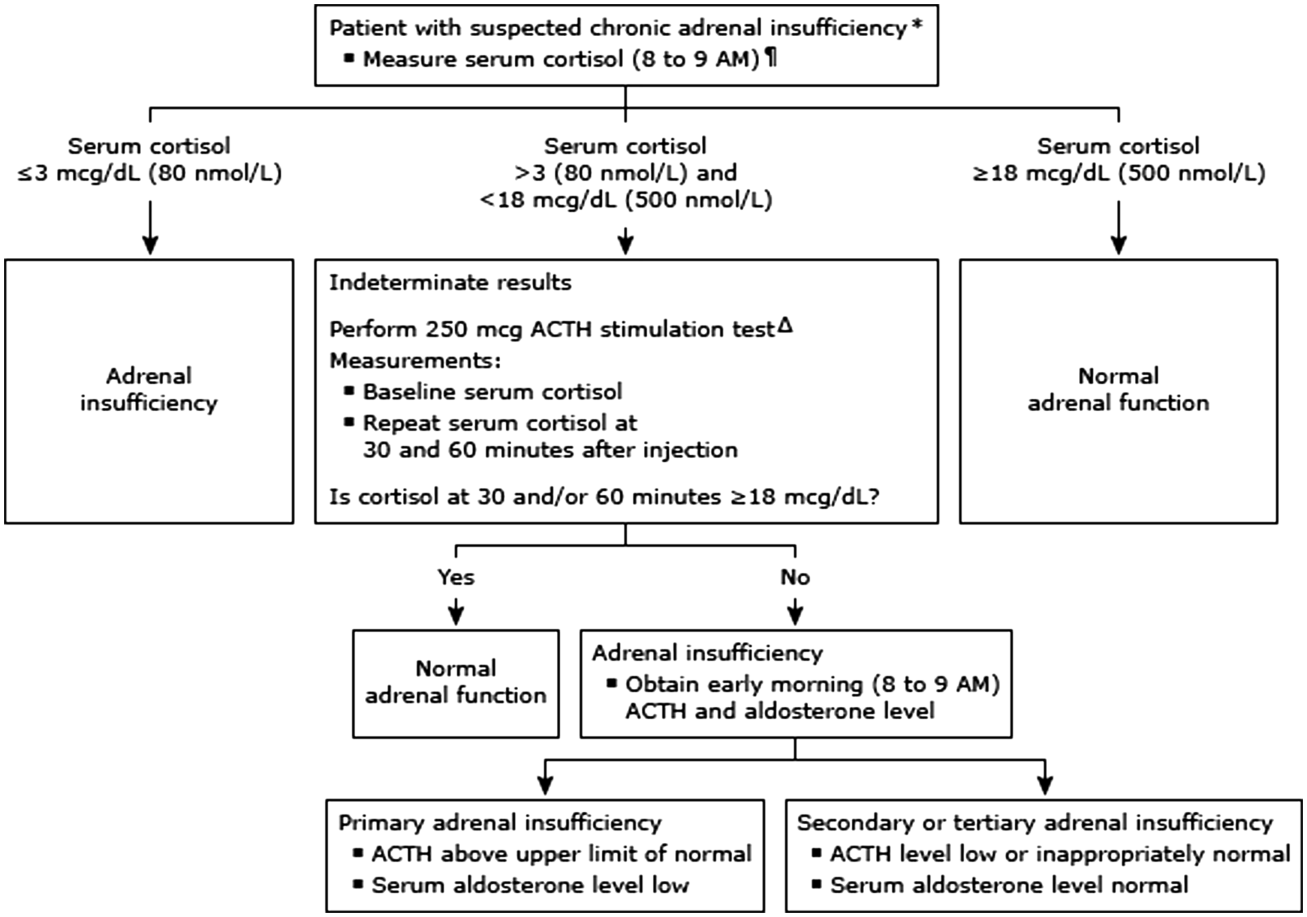
Diagnostic approach to suspected adrenal insufficiency (algorithm from UpToDate: diagnosis of adrenal insufficiency in adults).

To assess the proper functioning of the HPA axis, a 250-microgram ACTH stimulation test was conducted, with algorithm shown in Figure 2 (5). The results showed that aldosterone secretion function was intact, and that plasma cortisol levels increased from 11.17 μg/dL at baseline to 14.97 μg/dL at 30 min, and 17.95 μg/dL at 60 min, after ACTH was injected intravenously. Since a plasma cortisol concentration of at least 18 μg/dL after ACTH injection is considered normal, the results suggested partial or recent hypopituitarism, rendering the atrophy of the adrenal gland an inadequate cortisol response in this case (7). In addition, a magnetic resonance imaging (MRI) scan of the brain showed no abnormalities in the pituitary or hypothalamus glands, nor any new infarction or hemorrhage.

**Figure 3:**
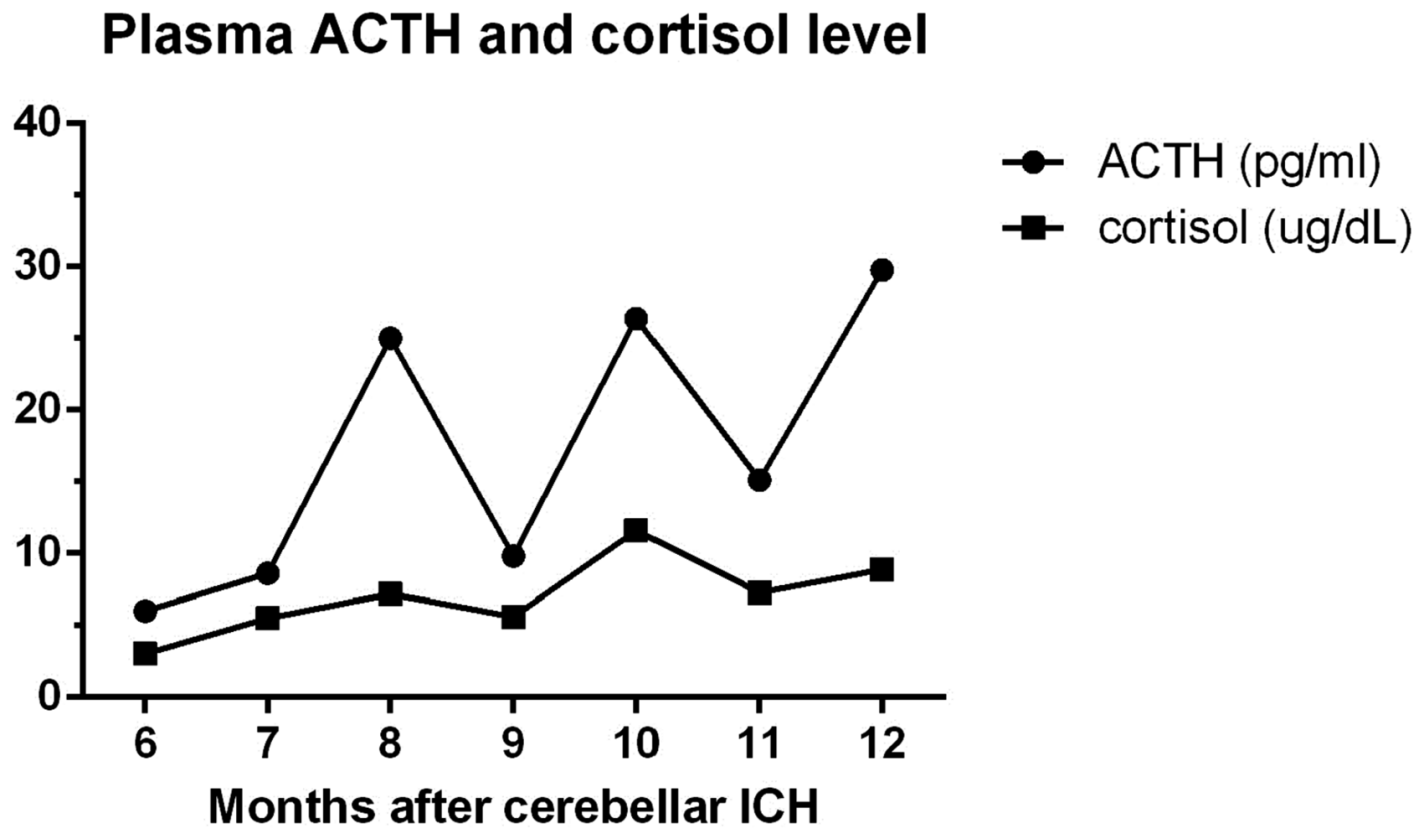
The patient’s plasma ACTH and cortisol levels were monitored from initial diagnosis of adrenal insufficiency (6 months after cerebellar ICH) to his discharge (12th month). ACTH: adrenocorticotropic hormone; reference range: 10–60 pg/mL; cortisol: 5–25 μg/dL.

Treatment was initiated using oral prednisone at a daily dose of 5 mg for the patient, and his clinical symptoms were evaluated regularly, with morning plasma ACTH and monthly cortisol level measurements (Figure 3). Based on these results, the dosage of the steroid used was gradually reduced. Although there were fluctuations in his plasma cortisol and ACTH levels, both the overall trend and his clinical symptoms improved over time. The patient’s blood pressure was consistently controlled throughout his hospital stay, and there were no recurrent episodes of fainting. Following 6 months of rehabilitation, the patient reported a marked reduction in symptoms such as weakness and dizziness, with minimal residual effects from the cerebellar ICH, and did not require further steroid treatment.

## Discussion

While there are documented case reports regarding the occurrence of adrenal insufficiency in critically ill patients in the relevant literature (3, 8), this report is the first known case of adrenal insufficiency associated with cerebellar ICH. ICH-related damage to the cerebellum, which plays a crucial role in voluntary muscle activation and coordination, often leads to balance disorders. These disorders can manifest as dizziness or vertigo, and may also be triggered by environmental changes, or changes in posture or motion. Dizziness is the most common complication of cerebellar ICH, leading physicians to overlook the possibility of other abnormalities, such as underlying endocrine issues.

Previous studies have shown that critical illness may occasionally result in suppressed plasma ACTH levels, in a condition known as critical illness-related corticosteroid insufficiency (CIRCI) (1). Cortisol levels are often increased by several folds during critical conditions, which was initially thought to be a result of elevated circulating ACTH. However, research has found that ACTH levels only briefly increase before returning to basal levels (2). The main cause of hypercortisolism in critical illness is reduced cortisol breakdown, rather than increased cortisol production (2, 9). Extended levels of elevated cortisol may affect the HPA axis by suppressing the release and production of plasma ACTH via negative feedback mechanisms. Over time, the absence of ACTH signaling to the adrenal gland results in a loss of integrity and function of the HPA axis (2, 8). According to the literature, the prevalence of adrenal insufficiency among critically ill medical patients is estimated to be between 10%–20%. Moreover, this rate increases to 60% in patients with septic shock (4). However, the prevalence of adrenal insufficiency following cerebellar ICH, and the average duration from a causal event to endocrinological recovery, remains elusive.

In this case study, the basal plasma ACTH level and ACTH stimulation test precluded the possibility of primary adrenal insufficiency because endogenous ACTH secretion would already be elevated and there would be little or no adrenal response to exogenous ACTH. The distinction between hypothalamic and pituitary causes of hypoadrenalism was made by determining the response to CRH. However, this is not typically necessary since their treatment is similar (i.e., replacement of glucocorticoids). In this case, the CRH stimulation test was not carried out. However, magnetic resonance imaging (MRI) of the brain was performed to effectively exclude any abnormalities in the pituitary and hypothalamus glands. While the definitive proof of the CRH stimulation test was lacking, the patient’s adrenal insufficiency was most likely secondary based on the present illness, monthly documented endocrinology data, and the explainable pathophysiology of CIRCI. It seemed compatible with the condition of a patient with clinical hypopituitarism and secondary adrenal insufficiency. While there may still be a detectable response, particularly if the hypopituitarism is partial and/or recent, the resultant adrenal dysfunction led to a subnormal cortisol response (5).

Although the overall trend of the patient’s plasma cortisol and ACTH levels were positively improving (Figure 3), fluctuation in the monthly intervals were noted. This was potentially related to our goal of achieving full recovery of the patient’s pituitary function. Therefore, if the patient’s endocrinological data showed improvements, glucocorticoid supplementation was gradually tapered, depending on his blood pressure and self-reported symptoms.

In conclusion, physicians must be aware of the associated symptoms of adrenal insufficiency in patients with cerebellar ICH and conduct the appropriate endocrinological tests to clarify the underlying disease, such as the ACTH stimulation test. When symptoms such as persistent dizziness, fatigue, or hypotension are present, an endocrine test is recommended. The patient’s status regarding adrenal integrity is important, as those with adrenal insufficiency should receive glucocorticoid replacement until the HPA axis recovers. In addition, to ensure better rehabilitation tolerance and functional outcomes, additional consideration when designing rehabilitation programs may be required to prevent accidents, such as falls. Further investigation is suggested to determine the prevalence of adrenal insufficiency following cerebrovascular accidents.

## Data availability statement

The raw data supporting the conclusions of this article will be made available by the authors, without undue reservation.

## Ethics statement

The Tri-Service General Hospital Ethics Review Board approved the data collection and case report publication with approval number A202105093. The studies were conducted in accordance with the local legislation and institutional requirements. The participants provided their written informed consent to participate in this study. Written informed consent was obtained from the individual(s) for the publication of any potentially identifiable images or data included in this article.

## Author contributions

Y-YL: Investigation, Writing – original draft. C-ML: Validation, Writing – review & editing. S-LC: Conceptualization, Supervision, Writing – review & editing.
